# Where empathy goes untranslated: the Chinese reflexive *Ziji* and the recoding of perspective in two complete English translations of *Hong Lou Meng*

**DOI:** 10.3389/fpsyg.2026.1948858

**Published:** 2026-09-30

**Authors:** Hongyu Zhu

**Affiliations:** Department of English (Education), Capital Normal University, Beijing, China

**Keywords:** empathy, *Hong Lou Meng*, literary translation, logophoricity, parallel corpus, point of view, reflexives, *Ziji*

## Abstract

The Mandarin reflexive *ziji* is a polyfunctional anaphor which, in logophoric uses, can also contribute to empathy and internal perspective. English *himself/herself* and *his/her own*, by contrast, are locally constrained and do not provide a general equivalent for this perspectival potential. The translation of *ziji* therefore offers a controlled window on the formal recoding of self-reference and on its possible consequences for viewpoint. Drawing on a purpose-built parallel corpus of 1,296 unique *ziji*-containing sentences from all 120 chapters of *Hong Lou Meng*, aligned with the Yang Xianyi and Gladys Yang translation and the Hawkes–Minford translation, this study annotates every source token for syntactic function and every target rendering for one of five strategies (reflexive, appositive intensifier, possessive own, lexicalization, and zero marking). Three principal findings emerge. First, ZERO (no overt reflexive or intensifying form at the *ziji* site) is the single most frequent outcome in both translations (63.5% for the Yangs; 49.1% for Hawkes–Minford); these rates measure local formal absence, not necessarily a loss of viewpoint. Second, zero rates track structural affordance: they peak where English grammar offers no categorial equivalent (subject position *ziji*) and fall where a direct equivalent exists (object position). A broad contextual cue—the presence of a preceding cognition, perception, or speech predicate—is not significantly associated with ZERO in either translation and is not treated as token-level evidence of logophoricity. Third, on a strictly parallel sub-corpus of 318 Chinese-English sentence pairs, Hawkes–Minford retain overt marking significantly more often than the Yangs (McNemar’s test, *p* < 0.001), reversing the conventional characterization of the Yangs as the more source-oriented translators. Qualitative case studies illustrate how viewpoint may be reduced, formally retained, or relocated through other discourse devices but do not estimate the corpus-wide incidence of these outcomes. The study argues that the perspectival consequences of translating *ziji* must be evaluated at the level of discourse rather than inferred from formal correspondence alone.

## 1 Introduction

Every reader of Chinese narrative prose knows the small word *ziji* (自己). Grammars label it a reflexive pronoun (Lyu and Kaiser, 2024; Sperlich, 2019), and in many sentences it behaves exactly as English *himself* does: it picks up a local antecedent in object position. But *ziji* leads a double life. It occurs freely as the subject of finite clauses, a position from which English reflexives are categorically barred; it can be bound across clause boundaries by an antecedent several clauses away; and, most importantly for this study, its long-distance uses cluster in environments of thought, perception, and speech, where the antecedent is the character from whose inner vantage point the discourse is presented. In such environments, *ziji* functions less as a device of coreference than as a marker of perspective: it signals that the narrating voice has momentarily merged with a character’s consciousness. It is, in Kuno’s terms, an empathy expression (Kuno and Kaburaki, 1977; Kuno, 1987), and in the terms of the logophoricity literature, an anaphor licensed by an attitude holder rather than by clause-mate syntax (Sells, 1987; Huang and Liu, 2001; Charnavel, 2020).

English has no single device that does all of this at once. Its reflexives are (largely) clause-bound; its intensifiers (*he himself*, *his own*) are emphatic rather than perspectival; and its principal resources for rendering a character’s inner vantage point, such as free indirect discourse, deictic alignment, and psych-verb framing, are structural and distributed rather than lexical and localized. Faced with *ziji*, then, the translator confronts a forced choice at every occurrence: reproduce the form, at the risk of unidiomatic emphasis, or reproduce the function, at the cost of the form. Multiplied across hundreds of occurrences in a long novel, these micro-choices aggregate into something larger: a systematic recoding, or a systematic erosion, of the empathetic texture of the original.

This study examines that aggregation empirically. Its material is *Hong Lou Meng* (also *The Dream of the Red Chamber*/*The Story of the Stone*), a novel whose narrative art depends to an unusual degree on rapid, unmarked shifts into and out of characters’ minds and which enjoys a rare privilege in translation studies: two complete, independent, and stylistically contrasting English translations—by Yang Xianyi and Gladys Yang (1978–1980) and by David Hawkes and John Minford (1973–1986). Working from a purpose-built parallel corpus of 1,296 *ziji*-containing source sentences spanning all 120 chapters, we ask three questions:

*RQ1*. By what formal strategies is *ziji* rendered in English, and in what proportions?

*RQ2*. How is the choice of strategy conditioned by the syntactic function of *ziji* in the source—that is, by whether English offers a categorial equivalent at that site?

*RQ3*. Do the two translations differ systematically, and if so, in which direction and with what consequences for the transfer of empathetic perspective?

The answers, we suggest, are of interest beyond *Hong Lou Meng* studies. Quantitatively, they show that in both translations, the modal formal outcome is no overt reflexive or intensifying form at the *ziji* site. This formal absence is patterned rather than random—it is most frequent where English grammar lacks a slot for the form, and its rate is significantly higher in the translation usually described as the more literal of the two. Qualitatively, the case studies show that viewpoint can be carried elsewhere even when no overt form appears at the *ziji* site: what Chinese encodes in a pronoun, English re-encodes in discourse architecture. The general lesson is that viewpoint equivalence between a ‘speaker-anchored’ language and a ‘structure-anchored’ one cannot be pursued form-by-form; it must be pursued at the level of the discourse unit, a conclusion with direct consequences for translation pedagogy and assessment in the Chinese-English pair.

## 2 *Ziji*, empathy, and point of view

### 2.1 *Ziji* beyond binding

The behaviour of *ziji* has been a proving ground for anaphora theory for four decades. Unlike English reflexives, bare *ziji* may take a long-distance antecedent across finite clause boundaries; it is subject-oriented; and its long-distance construal exhibits the well-known blocking effect, whereby an intervening first- or second-person subject blocks a more distant third-person antecedent (Cole et al., 1990; Pan, 1997; Huang, 2000), an effect experimentally confirmed and shown to be modulated by both syntactic prominence and perspective-taking (Lyu and Kaiser, 2021). A substantial line of analysis holds that long-distance *ziji* is not, strictly, a syntactic anaphor at all but a logophor: an element whose antecedent must be the bearer of the attitude, perception, or speech reported in its clause (Sells, 1987; Huang and Liu, 2001). On this view, long-distance *ziji* belongs with the dedicated logophoric pronouns of West African languages (Clements, 1975) and with ‘exempt’ uses of English reflexives (Charnavel, 2020; Zribi-Hertz, 1989): its licenser is a perspectival centre, not a clause-mate noun phrase (NP). That non-local *ziji* is sensitive to perspectival centres is now standardly acknowledged in the experimental literature as well (Lyu and Kaiser, 2021).

The pragmatic-discourse view is no longer sustained by theoretical argument alone. Sperlich (2016) shows that Korean learners of Chinese, transferring pragmatic anaphoric strategies from their L1, outperform English learners in interpreting long-distance *ziji*, as predicted if the form is pragmatically rather than syntactically regulated; and Zhang et al. (2021) report event-related potential (ERP) evidence that the referential resolution of ambiguous *ziji* is directly determined by discourse context, with discourse-level and sentence-level *ziji* anaphora recruiting distinct neurocognitive processes. Convergent electrophysiological evidence shows that, unlike the morphologically complex *ta-ziji*, whose retrieval is dominated by syntactic cues, bare *ziji* is retrieved with the guidance of semantic cues such as animacy (Zhu and Chai, 2025). Converging behavioural and neurocognitive evidence thus locates the interpretation of *ziji* in discourse—precisely the level at which, we will argue, its translation must also be assessed.

Three consequences of this literature matter for translation. First, *ziji* is polyfunctional: alongside the logophoric long-distance use, it occurs as a locally bound object (*shang hai ziji*, ‘hurt oneself’), as an adnominal possessive (*ziji de fang wu*, ‘one’s own house’), as an appositive intensifier after a pronoun or NP (*Baoyu ziji*, ‘Baoyu himself’), and crucially, as the overt subject of its own clause (*ziji men de*, ‘*ziji* was despondent’). Second, these functions map very unevenly onto English: object *ziji* has a near-perfect equivalent (*himself*); adnominal *ziji* is a good one (*his own*); intensifier *ziji* is an adequate one (*he himself*); but subject *ziji* has none, since English reflexives cannot be nominative subjects of finite clauses. Third, the perspectival load is not evenly spread across functions: it is concentrated in the long-distance and subject uses that arise within the scope of verbs of thinking, fearing, seeing, and saying—the uses English cannot calque.

### 2.2 Empathy and subjective construal

Kuno and Kaburaki (1977) introduced empathy as a grammatical primitive: the speaker’s identification, to varying degrees, with a participant in the reported event. Kuno (1987) develops a battery of empathy hierarchies and shows that reflexives in several languages are sensitive to them; Japanese *zibun*, the closest typological cousin of *ziji*, is the canonical case. Hirose (2000) refines the picture with the distinction between the ‘public self’ (the speaker as communicator) and the ‘private self’ (the speaker as thinking subject), arguing that Japanese *zibun* is the dedicated form of the private self, whereas English relegates the private self to derived contexts such as free indirect discourse. Ikegami (2008) generalizes the contrast as one of preferred ‘fashions of speaking’: languages such as Japanese favour subjective construal, in which the conceptualizer describes a scene from inside it, while English favours objective construal, in which the conceptualizer is displaced outside the scene (cf. Langacker, 2008). Although these proposals were developed largely for Japanese, the diagnostics carry over to Mandarin *ziji* in narrative: a sentence such as our Example (1) below presents Daiyu’s despondency from Daiyu’s own vantage, and *ziji* is the visible trace of that vantage.

For the full-corpus analysis, we code a binary contextual cue indicating whether *ziji* is preceded by a predicate of cognition, perception, or speech. This variable identifies environments compatible with logophoric licensing. It is used to explore whether this broad contextual configuration is associated with target-language realization. Claims concerning the preservation, relocation, or reduction of viewpoint are restricted to the qualitative case studies and are not extrapolated as corpus-wide frequencies.

### 2.3 Point of view in translation

The stylistic apparatus for describing point of view was assembled by Uspensky (1973), Fowler (1986), and Simpson (1993), who distinguished its phraseological, psychological, spatio-temporal, and ideological planes. That apparatus is still being extended. Shuttleworth (2024) draws on narrative theory, supplemented by Bakhtin’s notion of polyphony, to theorize how perspective is constructed and conveyed in translation and demonstrates that the question is not confined to literary narrative: even in encyclopedic prose that claims neutrality, translation choices can shift the perspective a text encodes. A corpus-based line of research has traced what becomes of these planes in translation. Bosseaux (2007) documents the erosion of free indirect discourse in French translations of Woolf; Mason and Şerban (2003) find deictic flattening in Romanian-English literary translation; and Munday (2008) shows how much ideological weight small shifts can carry.

Two generalizations emerge. First, viewpoint devices are fragile in translation: they are language-specific, and normalization removes them easily. Second, their loss is cumulative and largely invisible sentence by sentence, which is precisely why corpus methods are needed to detect it. Both generalizations, however, rest on European language pairs, in which point of view is carried configurationally—by free indirect discourse, deixis, and modality—rather than by a dedicated lexical item. What happens when the source language encodes perspective in a single morpheme remains open.

*Hong Lou Meng* itself has a rich tradition of corpus-based translation research, much of it comparing the Yang and Hawkes–Minford versions at the level of lexis, idiom, and culture-specific items (e.g., Li et al., 2011; Zhu and Miao, 2026). The received characterization in this literature is directional: the Yangs, translating at the Foreign Languages Press with a mandate of cultural fidelity, are held to be source-oriented and comparatively literal; Hawkes and Minford, writing for Penguin, are reader-oriented and comparatively free (Chou and Liu, 2024; Kwok et al., 2024; Zheng and Fan, 2023). Reflexivity and perspective, however, have not, to our knowledge, been examined systematically in this pair, and we will see that on this dimension the received characterization fails in an instructive way.

## 3 The study

### 3.1 Corpus

The cleaned corpus consists of 1,296 unique source sentences containing *ziji*, drawn from all 120 chapters of *Hong Lou Meng* and aligned at sentence level with their renderings in (i) *A Dream of Red Mansions*, translated by Yang Xianyi and Gladys Yang (Cao and Gao, 1978-1980; henceforth the Yangs translation), and (ii) *The Story of the Stone*, translated by David Hawkes (chapters 1-80; Cao, 1973-1980) and John Minford (chapters 81-120; Cao and Gao, 1982-1986; henceforth the Hawkes-Minford translation). Because Minford completed the Penguin version begun by his father-in-law and collaborator, and because the two volumes were produced within a single editorial project and stylistic frame, we treat Hawkes–Minford as one target text while reporting chapter ranges where relevant. The two translations rest on different base texts. The Yangs worked principally from a Youzheng text for the first eighty chapters and a Chengyi text for the last forty, while Hawkes and Minford worked chiefly from the Chengyi text. Sentences whose base texts diverge to the point of non-correspondence were excluded during compilation.

The design yields three analytic sets: a Yang set of 813 *ziji* sentences with their Yangs’ renderings; a Hawkes–Minford set of 803 sentences with their Hawkes–Minford renderings; and, embedded within these, a strictly parallel set of 320 sentences (all from chapters 81–120) for which both translations derive from the same source sentence. The full sets support distributional analysis of each translation; the parallel set supports direct, source-controlled comparison between translators (Section 4.3). A small number of source sentences lack an aligned rendering altogether: 3 in the Yang set and 7 in the Hawkes–Minford set (the latter including the passage in chapter 63 omitted from Hawkes’s translation). These gapped items are retained in the corpus totals above but excluded from all subsequent quantification, leaving 810 Yang and 801 Hawkes–Minford sentence pairs for analysis. Two of the gaps fall within the parallel set, which accordingly comprises 318 fully paired sentences for the between-translator comparison. Five exact duplicates Hawkes–Minford records in chapters 1–80 were identified during final cleaning and removed before all counts and tests reported below.

### 3.2 Annotation scheme

Each source sentence was annotated for the syntactic function of its (first) *ziji* token in four categories: subject position (SUBJ), *ziji* as a clausal subject or post-adverbial subject of a predicate (the position with no English categorial equivalent); object position (OBJ), *ziji* as the object of a verb or preposition; adnominal (POSS), *ziji (de)* modifying a noun; and an appositive intensifier (INT), appositive intensifier following a pronoun or NP. The number of *ziji* tokens per sentence was recorded, as was a binary contextual cue marking whether a predicate of cognition, perception, or speech precedes *ziji* in the sentence. The latter is a distributional property of the sentence, not a manual classification of the focal token as logophoric or empathetic.

Each target rendering was annotated for its treatment of *ziji* in five categories: REFL, an English reflexive pronoun in argument or adjunct position (*laughing at himself*, *kept to himself*); INTENS, an appositive intensifier (*he himself*); OWN, the possessive intensifier (*his own*); LEX, lexicalization of the reflexive meaning by an adverbial or phrase (*alone*, *in person*, *of his own accord*, *on his own*); and ZERO, the absence of any overt reflexive or intensifying element at the corresponding site—whether because a plain pronoun stands in, because the clause has been restructured, or because the material has been condensed away. ZERO therefore describes formal realization at the site, not whether the sentence is untranslated or whether the character’s viewpoint is preserved elsewhere. Accordingly, the five-way scheme is intentionally designed to answer this study’s primary question—whether *ziji* receives overt marking at the corresponding target site—rather than to provide a second-stage taxonomy of all formally zero outcomes. Where a rendering contained more than one candidate device, the annotation recorded the device corresponding to the *ziji* site, with additional devices noted. The REFL/INTENS boundary was drawn by adjacency: a reflexive form was coded INTENS only where it immediately follows the noun phrase it intensifies (*Baoyu himself*); clause-final floating intensifiers separated from their antecedent by an object (*fold it himself*) were coded REFL, in line with the treatment of adjunct reflexives.

All 1,301 items were pre-annotated automatically (part-of-speech-based rules for source function; pattern matching for target strategy) and then verified and corrected manually by the first author against the full sentence pair. The complete initial set was independently verified by a second coder, a doctoral researcher in linguistics. The two coders agreed on 1,292 of 1,301 records (99.3%); under any allocation of the nine disagreements across the two annotation layers, Cohen’s κ ≥ 0.99 for both source function and target strategy. Five exact duplicate records were subsequently removed, yielding the final corpus of 1,296 unique items without changing any annotation decision.

### 3.3 Analysis

Quantitative analysis proceeds in four steps. The first three match the research questions: overall strategy distributions per translation (RQ1); strategy conditioned by source function, with Pearson chi-square tests on the SUBJ/OBJ contrast as the sharpest test of the structural-affordance hypothesis (RQ2); and translator comparison on the parallel set, using a chi-square test on the full strategy distributions and McNemar’s test with continuity correction on the paired overt-vs-zero outcomes (RQ3). Fourth, to test the empathy annotation directly, we cross-tabulated the binary contextual feature (+/−empathetic) with ZERO versus overt rendering for each translation and used Pearson chi-square tests, reporting odds ratios (ORs) with 95% confidence intervals. Qualitative analysis (Section 4.4) then examines representative sentence pairs to identify what the formal categories cannot demonstrate: where the empathetic function of a zero-marked *ziji* may have been relocated to other devices and where the surrounding discourse supports a genuine reduction of viewpoint. Across all principal tests, effect sizes are reported as odds ratios (ORs) with 95% confidence intervals for binary contrasts, Cramér’s *V* for the multi-category contingency comparison, and a matched-pairs odds ratio with a 95% confidence interval for the McNemar comparison; confidence intervals for odds ratios were calculated on the log-odds scale.

## 4 Results

### 4.1 The modal fate of *ziji* is zero

Table 1 gives the distribution of target strategies across the two cleaned full sets. The headline result is unchanged: in both translations, ZERO is by far the most frequent single outcome. The Yangs translation leaves 63.5% of *ziji* sites without any overt reflexive or intensifying element; Hawkes–Minford, 49.1%. Among overt strategies, the plain reflexive (REFL) leads in both translations, followed by possessive *own*; appositive intensifiers and lexicalizations are marginal.

**Table 1:** Rendering strategies for *ziji* in the two translations (full sets).

Strategy	Yangs *n*	Yangs %	Hawkes–Minford *n*	Hawkes–Minford %
REFL (himself, etc.)	153	18.9	224	28.1
INTENS (he himself)	20	2.5	37	4.6
OWN (his own)	113	14	137	17.2
LEX (alone, in person…)	10	1.2	7	0.9
ZERO	514	63.5	391	49.1
Total	810	100	796	100

Against the background sketched in Section 2, this is the quantitative face of a typological mismatch. *Ziji* averages more than 10 occurrences per chapter in our extraction; at roughly half to two-thirds of those sites, no overt reflexive or intensifying form appears at the *ziji* site. This count establishes local formal absence only: a plain pronoun, restructuring, or condensation may carry some or all of the relevant meaning elsewhere. Whether ZERO also entails a loss of empathetic viewpoint cannot be inferred from formal absence alone. Sections 4.2–4.4 therefore separate the statistically demonstrated structural and contextual patterns from qualitative interpretation of what, if anything, compensates for the missing form.

### 4.2 Zero marking tracks structural affordance

Table 2 breaks the ZERO rate down by the syntactic function of the source token and reports the raw ZERO counts beside the percentages. The ordering is identical in both translations and matches the availability of an English categorial equivalent point for point. Zero marking peaks for SUBJ *ziji* (68.1% Yangs; 53.0% Hawkes–Minford)—the one position for which English grammar supplies no reflexive form at all—and bottoms out for OBJ *ziji* (52.8%; 36.3%), the position where himself is a drop-in equivalent. The SUBJ–OBJ contrast is significant in both translations. In the Yangs translation, the odds of ZERO realization are 1.91 times higher for SUBJ than for OBJ tokens (*χ*^2^(1) = 7.50, *p* = 0.006, OR = 1.91, 95% CI [1.20, 3.04]). The corresponding odds ratio for Hawkes–Minford is 1.99 (*χ*^2^(1) = 8.33, *p* = 0.004, OR = 1.99, 95% CI [1.24, 3.18]). POSS and INT fall in between, and their overt renderings gravitate to the predicted dedicated forms: OWN is the leading overt strategy for POSS in both translations (26.0 and 25.5% of POSS tokens, respectively), while INT attracts the highest INTENS rates (8.6 and 8.1%). Figure 1 visualizes the identical SUBJ > POSS > INT > OBJ gradient in ZERO rates across the two translations.

**Table 2:** ZERO rate by syntactic function of source *ziji.*

Function of *ziji*	Yangs *n*	Yangs ZERO *n* (%)	Hawkes–Minford *n*	Hawkes–Minford ZERO *n* (%)
SUBJ (subject position)	401	273 (68.1%)	394	209 (53.0%)
POSS (adnominal)	215	136 (63.3%)	212	109 (51.4%)
INT (appositive intensifier)	105	58 (55.2%)	99	40 (40.4%)
OBJ (object position)	89	47 (52.8%)	91	33 (36.3%)
Total	810	514 (63.5%)	796	391 (49.1%)

**Figure 1:**
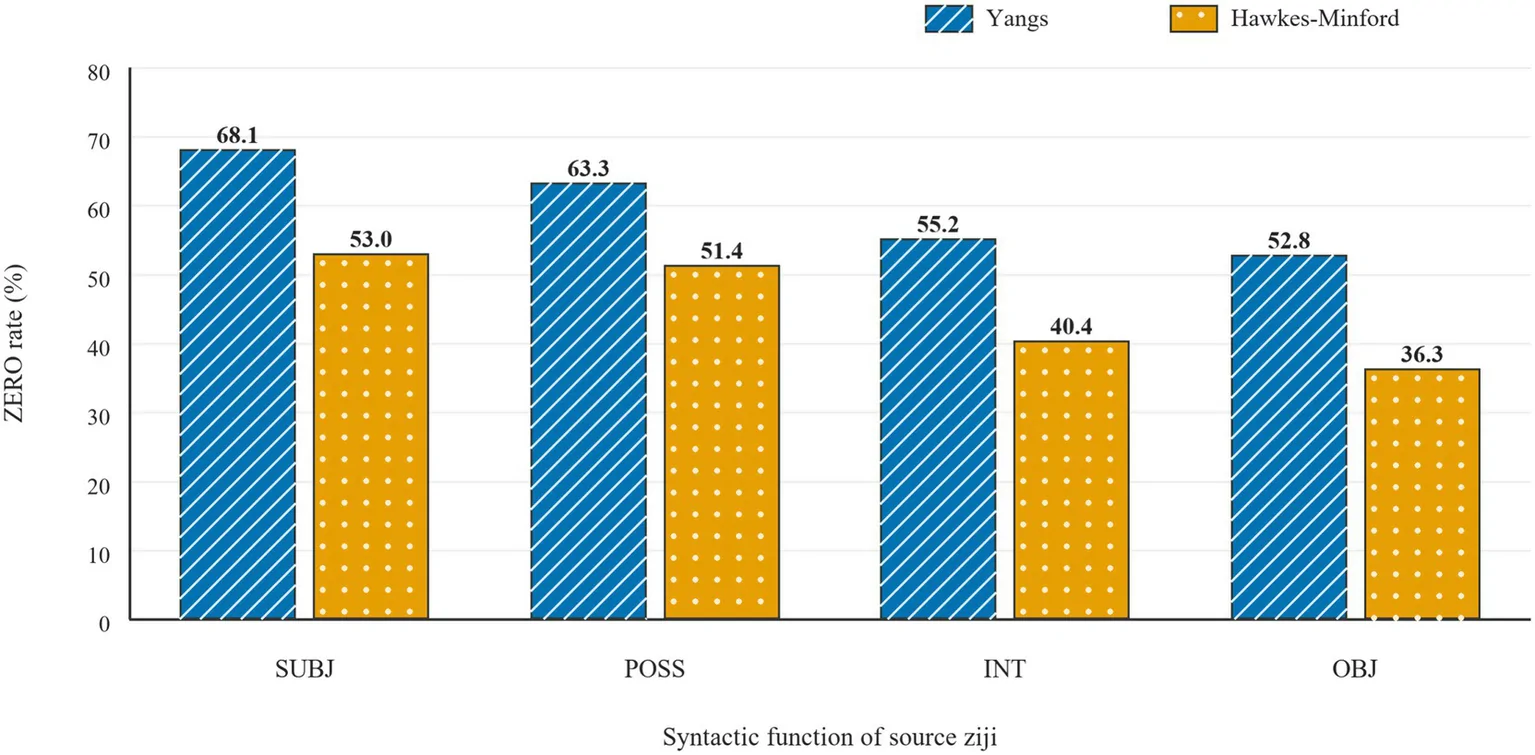
ZERO rates by syntactic function in the two translations.

The contextual annotation provides a direct test of the relationship between the distributional empathy proxy and formal realization (Table 3). In the Yangs translation, ZERO occurred in 304 of 489 + empathetic contexts (62.2%), compared with 210 of 321 empathetic contexts (65.4%; *χ*^2^(1) = 0.88, *p* = 0.347, OR = 0.87, 95% CI [0.65, 1.17]). The corresponding rates in Hawkes–Minford were 240 of 479 (50.1%) and 151 of 317 (47.6%; *χ*^2^(1) = 0.47, *p* = 0.495, OR = 1.10, 95% CI [0.83, 1.47]). Neither association is significant. The binary contextual proxy therefore does not support a general claim that empathetic contexts are disproportionately zero-marked.

**Table 3:** ZERO realization by empathetic-context annotation.

Translation	+Empathetic ZERO *n* (%)	−Empathetic ZERO *n* (%)	OR (95% CI)	*χ*^2^(1)	*p*
Yangs	304/489 (62.2%)	210/321 (65.4%)	0.87 (0.65–1.17)	0.88	0.347
Hawkes–Minford	240/479 (50.1%)	151/317 (47.6%)	1.10 (0.83–1.47)	0.47	0.495

ORs compare the odds of ZERO realization in +empathetic relative to −empathetic contexts.

Two implications follow. First, translational zero here is strongly conditioned by grammar: where the target language has a category suited to the source function, translators use overt marking more often; where it has none, ZERO is recorded more often. Second, structural affordance and empathetic context must be kept analytically distinct. Table 2 establishes a robust function gradient, whereas Table 3 provides no evidence that the binary empathetic-context proxy independently raises the probability of ZERO. Because that proxy is deliberately coarse, the null result does not demonstrate that empathy is irrelevant to individual translations; it does mean that claims about empathy loss or relocation should be treated as interpretations of the qualitative cases rather than as a corpus-wide statistical tendency.

### 4.3 The translators differ in the unexpected direction

The full-set comparison in Table 1 already suggests that Hawkes–Minford mark *ziji* overtly more often than the Yangs, but the two full sets contain largely different source sentences. The parallel set of 318 fully paired sentences from chapters 81–120 removes that confound: here both teams translated the same Chinese sentences from closely corresponding base texts. Table 4 shows that the difference survives intact. On identical input, the Yangs zero-mark 65.1% of sites; Hawkes–Minford 50.9%; the full strategy distributions differ significantly (*χ*^2^(4) = 14.50, *p* = 0.006, Cramér’s *V* = 0.151). The paired analysis is sharper still: of the 109 pairs in which exactly one translation marks *ziji* overtly, it is Hawkes–Minford in 77 and the Yangs in 32 (McNemar’s *χ*^2^ = 17.76, *p* < 0.001; matched OR = 2.41, 95% CI [1.59, 3.63]). Whatever the received wisdom about the Yangs’ literalism, on this variable it is the ‘free’ translation that stays closer to the surface of the source.

**Table 4:** Strategy distributions on the strictly parallel set (*n* = 318 sentence pairs, chapters 81–120).

Strategy	Yangs *n*	Yangs %	Hawkes–Minford n	Hawkes–Minford %
REFL	60	18.9	81	25.5
INTENS	8	2.5	16	5.0
OWN	38	11.9	55	17.3
LEX	5	1.6	4	1.3
ZERO	207	65.1	162	50.9

A caveat is in order: the parallel set falls in the Minford-translated chapters, so the comparison is strictly Yangs versus Minford. Three considerations mitigate the worry. The cleaned full-set ZERO rates for the Hawkes chapters (47.8% in chapters 1–80) and the Minford chapters (51.1% in chapters 81–120) run in the same direction and at similar magnitude; Minford worked within Hawkes’s established stylistic frame and completed the project as his collaborator; and the translator-internal control on the Yang side—whose ZERO rate is 62.3% in chapters 1–80 and 65.2% in chapters 81–120—shows that the variable is reasonably stable within a translation team across the textual seam. We therefore read the contrast as a property of the two projects while flagging the attribution issue for future studies on a chapter-matched Hawkes sample.

How does the asymmetry arise? Not principally through disagreement about which strategy to use when both mark: in the 79 pairs where both translations are overt, they choose the same category in 49 of these. It arises through Hawkes–Minford marking, where the Yangs stay silent. This is consistent with a well-documented global property of the two texts: the Hawkes–Minford version is substantially longer and more explicit than its source and than the Yangs version (Li et al., 2011), a profile that corpus-based translation studies would describe as explicitating (Baker, 1996; Blum-Kulka, 1986). On the evidence here, reflexive and perspectival marking is one of the things Hawkes–Minford explicitates: where the Chinese has an empathy-laden *ziji* and the Yangs write a plain pronoun, Hawkes–Minford reaches for *himself*, *he himself*, or *his own*—devices which, although not perspectival in the Chinese sense, at least keep the referent’s self-relation on the surface of the text.

### 4.4 Where the empathy goes: qualitative evidence

Numbers establish that *ziji* is mostly silenced and differentially silenced; they cannot demonstrate what the silence costs or what compensates for it. The purpose of the following analysis is not to estimate the corpus-wide frequencies of ZERO subtypes, which would require a new token-level coding instrument and an independent reliability assessment. Rather, the cases partially unpack the pooled category by showing that plain-pronoun replacement, restructuring or condensation, and discourse-level relocation can have different consequences for viewpoint. This section examines representative cases (back-translations are ours; *ziji* and its correspondents are italicized).

*Loss: perspective reduction through restructuring*. Example (1) is the canonical case. The source presents Daiyu’s mood from inside: the chain *jian* ‘see’ … *ting jian* ‘hear’ … *ziji men de* ‘(and) *self* was despondent’ keeps the camera behind Daiyu’s eyes, with the subject *ziji* as the grammatical trace of that internal vantage.

(1) Source Text: 这里林黛玉见宝玉去了, 又听见众姊妹也不在房, *自己*闷闷的。 (ch. 23)

[Daiyu saw that Baoyu had gone and heard that the girls were not in their rooms either; *self* was despondent.]

Yangs: With Baoyu gone and the other girls all out too, Daiyu did not know what to do. [ZERO]

The English is accurate and fluent, and nothing in it is ‘wrong’. But the absolute construction *With Baoyu gone* reports the situation from outside; the perceptual verbs are gone, and with them the warrant for reading the despondency as experienced rather than observed. In this case, restructuring removes the perceptual frame and reduces the internal viewpoint. This illustrates one possible consequence of the 68.1% ZERO rate for SUBJ tokens in Table 2: not a statistically established loss in every case, but a recurrent potential for externalization that requires discourse-level examination.

*Preservation: own as a viewpoint holdout*. Where the function is adnominal, English *own* can carry a surprising amount of the load. In (2), the whole sentence sits inside the Lady Dowager’s assessment (*jian* ‘seeing’, *liao* ‘reckoning’), and *ziji shen bian* ‘by *self’s* side’ is bound by her across the clause:

(2) Source Text: 贾母见雪雁甚小……料黛玉皆不遂心省力的，便将*自己*身边一个二等丫头……与了黛玉。 (ch. 3)

[The Lady Dowager, seeing Xueyan so young … and reckoning that Daiyu would not be well served, gave Daiyu a second-grade maid from *self’s* side.]

Yangs: Since the Lady Dowager considered Xueyan too young and childish …, she gave Daiyu one of *her own* personal attendants … [OWN]

*Her own* is licensed here precisely because the matrix frame (*considered*) already establishes the Dowager as attitude holder; the possessive intensifier then re-anchors the maid to her sphere from her point of view. The 14.0–7.2% OWN rates in Table 1 are, on inspection, largely of this type: *own* functions as English’s most reliable, if partial, receptacle for empathetic *ziji*.

*Relocation: empathy re-coded in discourse structure*. The most interesting cases are those counted as ZERO or as routine REFL in which the perspectival study of *ziji* re-emerges elsewhere. In (3), Jia Rong, in bitter self-reproach, interrogates *ziji* while slapping his own face:

(3) Source Text: 又*自己*问着自己说:“以后可还再顾三不顾四的不了?” (ch. 68)

[And *self* asked *self*, saying: ‘In future will you still meddle so recklessly?’]

Hawkes–Minford: … prefacing each blow with an interrogation, thus: ‘Are you going to go on doing these stupid, meddlesome things in future?’ (slap!) [ZERO]

Formally, neither *ziji* token has an overt reflexive or intensifying form at its source-corresponding site. Functionally, Hawkes has rebuilt the self-address as dramatized second-person speech punctuated by stage directions—arguably a more vivid interiority than a literal ‘asked himself’ would give. Example (4) shows the same logic in the aggregate style of the Hawkes chapters: for *sheng de ziji luo dan* ‘left *self* all alone’, Hawkes spends two devices where the source spends one:

(4) Source Text: 却说宝玉因近日林黛玉回去, 剩得*自己*落单…… (ch. 13)

[Baoyu, because Daiyu had lately gone home, was left *self* all alone …].

Hawkes–Minford: … Bao-yu … had kept *to himself*, going to bed early every night and sleeping disconsolately *on his own*. [REFL + LEX]

The doubling (*to himself* … *on his own*), together with the evaluative adverb *disconsolately*, does with lexical accumulation what *ziji* does with a pronoun: it holds the paragraph inside Baoyu’s loneliness. This explicitating habit is the qualitative face of the McNemar asymmetry in Section 4.3.

*Divergence on identical input*. The parallel set lets us watch the two teams solve the same problem. In (5), Hua Zifang shows his sister the trousseau *ziji* prepared; the Yangs translate the referential content with a bare pronoun, whereas Minford spends two intensifiers to keep the brother’s personal agency in view:

(5) Source Text: 又把*自己*所办妆奁一一指给他瞧…… (ch. 120)

[… and pointed out to her one by one the trousseau *self* had prepared …].

Yangs: … as well as the dowry he had prepared … [ZERO]

Hawkes–Minford: … and the trousseau that *he himself* had made ready for her … a part he had provided *himself*. [INTENS + REFL].

Finally, (6) shows that both translations overtly render the possessive *ziji* with *own*. The contrast lies in information structure rather than zero marking: the Yangs make Shiyin the grammatical subject and background Yucun’s desire to continue questioning, whereas Minford foregrounds Yucun as an experiencer through curiousity and wanting. The difference is one of perspectival prominence, not complete preservation versus loss.

(6) Source Text: 食毕, 雨村还要问*自己*的终身。 (ch. 120)

[The meal over, Yucun still wanted to ask about *self’s* future.]

Yangs: After the meal, Shiyin forestalled further questions about Yucun’s *own* future. [viewpoint rotated to Shiyin]

Hawkes–Minford: When they had finished their meal, Yu-cun was still curious, this time wanting to know the secrets of *his own* future … [OWN, viewpoint retained].

Across the case studies, then, three possible fates of empathetic *ziji* can be distinguished beneath the strategy counts: perspective reduction (1), containment in a dedicated but weaker English form (2, 5, 6 Hawkes–Minford), and relocation into discourse architecture—direct speech, free-indirect colouring, psych-verb framing, and lexical accumulation (3, 4). The quantitative results establish that ZERO is common, especially in the Yangs translation and in the subject position; the non-significant contextual tests in Table 3 mean that they do not establish how often ZERO amounts to empathy loss. That further inference rests on the qualitative evidence.

Taken together, the case studies show that formal coding and perspectival effects do not map onto each other in a one-to-one fashion. Example (1) illustrates the local compression of perceptual framing, although the surrounding translation continues to represent Daiyu’s mental experience. Examples (2) and (6) retain possessive self-reference through *own*, while Example (5) illustrates translator divergence in the overt marking of personal agency rather than unambiguous preservation of empathy. Examples (3) and (4) show how meanings associated with self-relation or solitude may be redistributed through direct speech, syntactic restructuring, and lexical elaboration; such redistribution does not by itself demonstrate either the preservation or the loss of interior viewpoint.

## 5 Discussion

### 5.1 A typological asymmetry in the coding of perspective

The results give empirical substance to the contrast sketched in Section 2.2, while also delimiting its empathetic interpretation. Chinese, like Japanese, possesses a dedicated, lexicalized carrier of the private self: a single word that can, in one breath, both corefer and empathize. English distributes comparable study over structural means (free indirect discourse, deictic and temporal alignment, psych-verb framing, and information structure), none of which is triggered automatically by anything in the source sentence. The consequence for translation is asymmetric by design. A form-driven translator working from Chinese into English finds no landing place for the form at roughly half of all *ziji* sites and at two-thirds of the empathetically crucial subject sites; the site may remain formally unmarked, or the translator may rebuild the perspective by other means. Table 3, however, shows that the binary empathetic-context proxy does not significantly increase ZERO realization in either translation. The zero rates therefore measure formal absence, whereas the claim that viewpoint is lost or relocated requires discourse-level interpretation.

The structural-affordance gradient in Table 2 is what makes this a grammatical finding rather than merely a stylistic one. If zero marking reflected nothing but translatorial taste, there would be no reason for both translations to reproduce the same ordering, SUBJ > POSS > INT > OBJ, which is precisely the ordering of decreasing distance from an available English category. Zero rendering is here the visible footprint of a typological gap, and the *Hong Lou Meng* corpus, with its two independent target texts, in effect replicates the observation once per translation team.

### 5.2 Rethinking the literal/free contrast

The translator effect runs against the standard characterization of this famous pair. On the dimension of reflexive and perspectival marking, it is Hawkes–Minford, the supposedly domesticating and reader-oriented version, who keeps the source’s self-marking on the surface, whereas it is the Yangs, the supposedly faithful version, who normalize it away. The paradox dissolves once ‘literal’ is unpacked. The Yangs’ fidelity operates at the level of propositional and cultural content, and their prose is economical; a redundant *himself* is exactly the kind of expendable form that such economy prunes. Hawkes–Minford’s freedom operates at the level of sentence architecture, and its engine is explicitation: they routinely spend more words than the source, and some of those words are *himself*, *he himself*, and *his own*. The finding therefore refines the received view rather than simply contradicting it. Source-orientation and target-orientation are not scalar opposites but bundles of level-specific habits, and a variable such as *ziji*-marking can align with those bundles in the direction opposite to the one the labels predict. The finding also contributes a perspectival case study to the explicitation literature (Blum-Kulka, 1986; Baker, 1996): what is made explicit here is neither connectives nor encyclopaedic content, but the self-relation of a character, which is to say viewpoint itself.

### 5.3 Implications for translating viewpoint between Chinese and English

Three practical maxims follow. First, in Chinese-to-English translation, *ziji* should be read before it is rendered: the translator’s diagnostic question is not ‘reflexive or intensifier?’ but ‘whose consciousness is this clause in?’ Where the answer is a character’s, the absence of overt marking at the *ziji* site is acceptable only if the perspective is paid for elsewhere in the discourse unit, whether by free indirect discourse, by retaining the perceptual and psych-verbs that license it, or by devices of the kind seen in Examples (3), (4), and (6). Second, *own* deserves promotion in the translator’s toolkit: it is the one English form that couples nominal syntax with a genuinely perspectival flavour, and the corpus shows both teams instinctively reserving it for empathetic adnominal *ziji*. Third, in the reverse direction, English-to-Chinese translators should be alert to the mirror-image trap: English free indirect discourse carries no lexical flag, and a Chinese rendering that fails to plant *ziji* (and related empathy expressions) at the right sites will read as externally narrated even when every proposition is faithfully conveyed. In both directions, the operative unit of viewpoint equivalence is the discourse span, not the sentence.

## 6 Conclusion

This study set out to establish what happens to the empathetic viewpoint encoded in Mandarin *ziji* when *Hong Lou Meng* is translated into English. Across 1,296 unique source sentences and two complete translations, the answer is threefold.

Formally, the modal outcome is ZERO: at roughly half to two-thirds of *ziji* sites, the English text carries no overt reflexive or intensifying form at the corresponding site, and this formal absence is most frequent for subject-position *ziji*, where English lacks a reflexive subject form. Structurally, this formal absence is grammatically patterned, tracking the availability of English categorial equivalents with identical gradients in both translations. The binary empathetic-context proxy, however, is not significantly associated with ZERO in either translation; the corpus-wide statistics therefore establish formal and structural patterning, not a general rate of empathy loss. Translationally, the two versions divide in a direction that the literal/free stereotype does not predict: on identical input, Hawkes–Minford preserve overt self-marking significantly more often than the Yangs, an explicitating habit that retains at least a formal trace of the source’s perspectival texture. The qualitative record shows that formal preservation and perspectival preservation are separable achievements. Empathy can survive zero marking when it is relocated into direct speech, psych-verb framing, or free indirect discourse, and it can wither behind a dutiful himself.

Each of these findings comes with a boundary that also marks out the work still to be done. The formal result rests on a category, ZERO, that is heterogeneous by design. Section 4.4 partially addresses this heterogeneity by distinguishing representative cases of plain-pronoun substitution, clausal or discourse restructuring, condensation, and the reduction or relocation of viewpoint. A corpus-wide quantitative decomposition would, however, require a new token-level coding instrument, explicit decision rules for borderline cases, and an independent reliability assessment. Because that analysis asks a distinct question—how formally zero renderings are internally distributed and which subtypes preserve, relocate, or reduce perspective—we reserve it for a dedicated follow-up study building on this corpus. The contextual analysis uses a deliberately distributional proxy—whether cognition, perception, or speech precedes *ziji*—and its null result does not exclude effects for more finely distinguished types of logophoric licensing; token-level pragmatic coding with independent reliability assessment would provide a sharper test. The translational result draws its strongest evidence from a strictly parallel set that lies in the Minford chapters, so the comparison inherits an attribution caveat, mitigated but not eliminated by the full-set and translator-internal controls of Section 4.3; a chapter-matched Hawkes sample would settle it. Beyond these, this study concerns one novel, in one genre, in one language pair, and the structural-affordance logic predicts comparable gradients for Japanese *zibun* and Korean *caki* in English translation. That prediction is the most obvious extension of this study’s design, and it is testable with the same instrument.

For translation studies, then, the case of *ziji* argues that point of view must be audited at the level of discourse rather than the sentence and that source-orientation and target-orientation are bundles of level-specific habits rather than positions on a single scale. In this respect our conclusion converges with earlier studies arguing that *ziji* is regulated by discourse-pragmatic rather than purely syntactic principles (Sperlich, 2016; Zhang et al., 2021): the meaning of *ziji* is a function of the discourse in which it is embedded, and it is by reading that discourse—whose consciousness the clause inhabits, what attitude predicate licenses the form—that its interpretation, and hence its translation, is to be recovered. What those studies establish for the interpretation of *ziji*, this study extends to its rendering: a form whose meaning lives in discourse can only be translated at the level of discourse. For linguistic typology, finally, the case offers translation corpora as a natural experiment in how languages that construe scenes from within are read by languages that construe them from without.

## Data Availability

The original contributions presented in the study are included in the article/supplementary material, further inquiries can be directed to the corresponding author/s.
